# Cesarean scar endometriosis: presentation of 198 cases and literature review

**DOI:** 10.1186/s12905-019-0711-8

**Published:** 2019-01-18

**Authors:** Ping Zhang, Yabing Sun, Chen Zhang, Yeping Yang, Linna Zhang, Ningling Wang, Hong Xu

**Affiliations:** 1 The International Peace Maternity and Child Health Hospital, School of Medicine, Shanghai Jiao Tong University, Shanghai, 200030 China; 2 Department of Assisted Reproduction, Shanghai Ninth People’s Hospital, School of Medicine, Shanghai Jiao Tong University, Shanghai, 200011 China

**Keywords:** Cesarean scar endometriosis, Abdominal wall endometriosis, Cesarean section, Pfannenstiel incision

## Abstract

**Background:**

Cesarean scar endometriosis (CSE) is the most common type of abdominal wall endometriosis (AWE). The aim of this study was to systematically identify the clinical features of CSE and recommend precautionary measures.

**Methods:**

A large, retrospective study was undertaken with CSE patients treated surgically at our hospital between January 2005 and December 2017.

**Results:**

A total of 198 CSE patients were enrolled, with a mean age of 32.0 ± 4.0 years. The main complaint of the patients was abdominal mass (98.5%), followed by cyclic pain (86.9%). The latency period of CSE was 31.6 ± 23.9 months, and the duration between the onset of symptoms and this surgery was 28.3 ± 25.0 months. A majority (80.8%, *n* = 160) of the patients had undergone a Pfannenstiel incision, and a minority (19.2%, *n* = 38) a vertical midline incision. The latency period of CSE in the case of a Pfannenstiel incision was significantly shorter than that in the case of a vertical midline incision (24.0 vs 33.0 months, *P* = 0.006). A total of 187 (94.4%) patients had a single endometrioma, 11 (5.6%) patients had multiple endometriomas, and the 11 multiple-endometrioma patients had all undergone a Pfannenstiel incision. Lesions of endometrioma were common in corner sites, after either incision: 142/171 (83.0%) in Pfannenstiel incision scars and 32/38 (84.2%) in vertical incision scars.

**Conclusions:**

The findings of this study indicate that the Pfannenstiel incision carries a higher risk of CSE than the vertical midline incision. Thorough cleaning at the conclusion of CS, particularly of both corner sites of the adipose layer and the fascia layer, is strongly recommended for CSE prevention. Further studies might provide additional recommendations.

## Background

Endometriosis is a sex hormone-dependent gynecological disease that is characterized by the growth of endometrial tissue outside the uterine cavity [1]. It usually occurs in the pelvis, at sites such as the ovaries and the pelvic peritoneum. However, ectopic endometrial tissue can also be found outside the pelvis, at sites such as the lung, brain, bowel, and abdominal wall [2–4]. The presence of ectopic endometrial tissue embedded in the subcutaneous adipose layer and the muscles of the abdominal wall is called abdominal wall endometriosis (AWE). AWE can occur spontaneously, but usually develops in association with a previous surgical procedure, such as a cesarean section (CS), hysterectomy, or appendectomy [5–7].

Cesarean scar endometriosis (CSE) is the most commonly reported type of AWE [8]. Nominato et al. suggested that CS greatly increased the risk of developing AWE [9]. The pathophysiology of CSE may be due to the direct implantation of endometrial tissue in the cesarean incision (the implantation theory) [8]. During cesarean delivery, the endometrial tissue is inoculated directly in the cesarean incision. With an appropriate supply of nutrients and hormonal stimuli, these endometrial cells survive and proliferate, which finally leads to CSE. Although it is an unusual disease, with a reported incidence of 0.03–0.45%, CSE may cause long-term discomfort involving cyclic lower abdominal pain [10, 11]. Case reports of malignant transformation of CSE have also been sporadically reported [12–14].

Generally, few publications have focused on CSE, and a majority of them are either case series or case reports [15–18]. As a large obstetric and gynecologic hospital, we have treated many CSE patients and were very interested in identifying the characteristics associated with CSE. Here, we report our findings in managing CSE over approximately the last decade, and we discuss the clinical features and prevention strategies of this rare disease.

## Methods

This retrospective study was conducted in the Department of Obstetrics and Gynecology of the International Peace Maternity and Child Health Hospital (IPMCH). The study was approved by the ethical review committee of the IPMCH.

This study included patients diagnosed with CSE who received surgical treatment at IPMCH between January 2005 and December 2017. The inclusion criteria were the following: (1) patient had a history of at least one CS and the symptoms occurred after the cesarean delivery; (2) surgical excision with surrounding clear margins was performed; and (3) pathological diagnosis for each excised lesion was endometriosis.

The baseline characteristics and surgical processes of all of the patients were recorded in the database of our hospital. We conducted a computerized search of the database according to the disease name. The preliminarily selected items were further checked and screened artificially following the inclusion criteria. The main symptoms of CSE included a palpable mass, under or away from the scar, with cyclic pain and swelling during menstruation. The latency period was defined as the time between CS and the onset of symptoms. The following information was extracted: age, age at CS, parity, delivery history, incision type, symptoms, size of the mass, latency period, duration between symptoms and surgery, operative findings, and histopathological evaluations.

The normality of continuous variables was tested using the Shapiro–Wilk test. The comparisons of normally distributed, continuous data were made with the Student *t* test and an analysis of variance. Two sets of nonnormally distributed, continuous data were analyzed with Mann–Whitney U tests; otherwise, Kruskal–Wallis H tests were employed. The categorical data were analyzed with χ^2^ and Fisher exact tests. The differences were considered statistically significant when the *P*-value was less than 0.05. Statistical analysis was performed using SPSS version 17.0 (SPSS Inc., Chicago, IL, USA).

## Results

Following the inclusion standards described above, a total of 198 CSE cases were ultimately enrolled and all the cases were pathologically confirmed. Baseline characteristics of the patients are summarized in Table 1. The mean age of the patients was 32.0 ± 4.0 years, and the mean age at the time of CS was 27.1 ± 3.5 years. Parity of the patients ranged from 1 to 2. All of the patients had a history of CS: 186 (93.9%) patients had 1 CS, and 12 (6.1%) had 2. The latency period of CSE ranged from 1 to 120 months, with a mean of 31.6 ± 23.9 months. The duration between the onset of symptoms and surgery was 28.3 ± 25.0 months.

**Table 1:** Baseline characteristics, symptoms, and CSE sites of the patients^a^

Characteristic	*n* (%)	Mean ± SD (range)
Age of the patients (years)		32.0 ± 4.0 (21–43)
Age at CS (years)		27.1 ± 3.5 (19–37)
Parity		
1	175 (88.4)	
2	23 (11.6)	
No. of CSs		
1	186 (93.9)	
2	12 (6.1)	
Latency period (months)		31.6 ± 23.9 (1–120)
Duration between symptoms and surgery (months)		28.3 ± 25.0 (1–180)
Symptoms		
Abdominal mass	195 (98.5)	
Cyclic pain	172 (86.9)	
Noncyclic pain	26 (13.1)	
Dysmenorrhea	64 (32.3)	
Incision type		
Pfannenstiel	160 (80.8)	
Vertical midline	38 (19.2)	
No. of endometriomas		
Single	187 (94.4)	
Multiple	11 (5.6)	
Endometrioma location		
Pfannenstiel incision		
No. of endometriomas	171 (81.8)	
Left corner	65 (38.0)	
Middle	29 (17.0)	
Right corner	77 (45.0)	
Vertical midline incision		
No. of endometriomas	38 (18.2)	
Upper corner	15 (39.5)	
Middle	6 (15.8)	
Lower corner	17 (44.7)	

^a^Values are given as mean ± SD or as number (percentage)

Symptoms and CSE sites of the study patients are also shown in Table 1. The most common symptom of the patients was a palpable painful abdominal mass under or adjacent to the incision, accompanied with either cyclic pain (86.9%, *n* = 172) or noncyclic pain (13.1%, *n* = 26). A majority (80.8%, *n* = 160) of the patients had undergone a Pfannenstiel incision, and a minority (19.2%, *n* = 38) a vertical midline incision. In the group of 198 study patients, 187 (94.4%) patients had a single endometrioma and 11 (5.6%) patients had multiple endometriomas. The 11 multiple-endometrioma patients had all undergone a Pfannenstiel incision. In total, 209 abdominal wall endometriomas were excised. Specifically, 171 and 38 endometriomas were excised from Pfannenstiel incision and vertical midline incision scars, respectively. A majority of the endometriomas were located in corner sites, after either incision: 142/171 (83.0%) in Pfannenstiel incision scars and 32/38 (84.2%) in vertical incision scars.

We introduced an “upper bound” and a “lower bound” to describe the locations of the endometriomas for the first time. The abdominal wall was divided into the adipose layer, the fascia layer, the muscular layer, and the peritoneal layer. The bladder was considered the deepest layer involved. As shown in Tables 2, 64.6% (*n* = 135) of the endometriomas were located between the adipose layer and the fascia layer; 14.8% (*n* = 31) were located between the adipose layer and the muscular layer. Of the 209 endometriomas, 16 (7.7%) invaded the peritoneum; 1 (0.5%) invaded into the abdominal cavity; and 2 (1.0%) invaded the bladder.

**Table 2:** Location of the endometriomas in the abdominal wall

		Lower bound					
		AL, *n* (%)	FL, *n* (%)	ML, *n* (%)	PL, *n* (%)	AC, *n* (%)	B, *n* (%)
Upper bound	AL	12 (5.7)	135 (64.6)	31 (14.8)	11 (5.3)	1 (0.5)	1 (0.5)
	FL	/	0 (0)	12 (5.7)	3 (1.4)	0 (0)	0 (0)
	ML	/	/	0 (0)	2 (1.0)	0 (0)	1 (0.5)

*AL* = adipose layer, *FL* = fascia layer, *ML* = muscular layer, *P* = peritoneum layer, *AC* = abdominal cavity, *B* = bladder

To identify the risk factors for CSE, we calculated the difference in latency period based on the patients’ baseline characteristics (Table 3). Age at CS, parity, previous CS, and dysmenorrhea showed no significant correlation with the latency period. Location of the endometriomas, such as under or away from the scar, or in the corner or in the middle of the scar, also showed no correlation with the latency period. However, the latency period showed significant correlation with the incision type, i.e., the latency period of the CSE in patients with Pfannenstiel incision was significantly shorter than that in patients with vertical midline incision (24.0 vs 33.0 months, *P* = 0.006). The location of the lower bound of the endometriomas also showed a correlation with the latency period (*P* = 0.011), i.e., the latency period was longer when the endometriomas invaded down to the peritoneum or the bladder.

**Table 3:** Difference in latency period based on patients’ baseline characteristics, symptoms, and CSE sites^a^

Characteristic	*n* (%)	Latency period (months)	*P*-value
		Median (quartiles)	
Age at CS (years)			0.941
≥ 35	6 (3.0)	30.0 (10.5–48.0)	
25–34	143 (72.2)	24.0 (12.0–40.0)	
≤ 24	49 (24.8)	24.0 (12.0–48.0)	
Parity			0.273
Nulliparous	178 (89.2)	24.0 (12.0–36.0)	
Multiparous	20 (10.1)	21.0 (6.0–48.0)	
One previous CS			0.452
Yes	21 (10.6)	24.0 (6.0–48.0)	
No	177 (89.4)	24.0 (12.0–36.0)	
Dysmenorrhea			0.473
Yes	64 (32.3)	19.0 (12.0–36.0)	
No	134 (67.7)	24.0 (12.0–36.0)	
Incision type			0.006
Pfannenstiel	160 (80.8)	24.0 (12.0–36.0)	
Vertical midline	38 (19.2)	33.0 (24.0–60.0)	
No. of endometriomas			0.078
Single	187 (94.4)	24.0 (12.0–48.0)	
Multiple	11 (5.6)	16.0 (12.0–24.0)	
Location of the endometriomas I			0.253
Under the scar	111 (56.1)	24.0 (12.0–36.0)	
Away from the scar	87 (43.9)	24.0 (12.0–48.0)	
Location of the endometriomas II			0.153
Corner of the scar	167 (84.3)	24.0 (12.0–48.0)	
Middle of the scar	31 (15.7)	30.0 (24.0–38.0)	
Upper bound of the endometriomas			0.073
Adipose layer	181 (91.4)	24.0 (12.0–39.0)	
Fascia layer	14 (7.1)	24.0 (18.0–49.5)	
Muscular layer	3 (1.5)	48.0 (48.0–56.0)	
Lower bound of the endometriomas			0.011
Adipose layer	10 (5.1)	27.0 (18.0–39.0)	
Fascia layer	130 (65.7)	24.0 (12.0–36.0)	
Muscular layer	41 (20.7)	24.0 (16.0–48.0)	
Peritoneum	12 (6.1)	48.0 (27.0–60.0)	
Abdominal cavity	3 (1.5)	30.0 (9.0–30.0)	
Bladder	2 (1.0)	40.0 (24.0–40.0)	

^a^Mann–Whitney U test for comparing two sets; Kruskal–Wallis H test for comparing multiple sets

To confirm the difference in latency period based on incision type, baseline characteristics of the patients with Pfannenstiel incisions or vertical midline incisions were further compared. As shown in Table 4, no significant difference was identified in the patients’ baseline characteristics.

**Table 4:** Comparison of the baseline characteristics between patients with Pfannenstiel incision or vertical midline incision^a^

Characteristic	Pfannenstiel	Vertical midline	*P*-value
	*n* (%)	*n* (%)	
Age at CS (years)			0.334
≥ 35	4 (2.5)	2 (5.2)	
25–34	119 (74.4)	24 (63.2)	
≤ 24	37 (23.1)	12 (31.6)	
Parity			0.195
Nulliparous	146 (73.7)	32 (16.2)	
Multiparous	14 (7.1)	6 (3.0)	
One previous CS			0.248
Yes	15 (7.6)	6 (3.0)	
No	145 (73.2)	32 (16.2)	
Dysmenorrhea			0.782
Yes	51 (25.8)	13 (6.6)	
No	109 (55.1)	25 (12.6)	

^a^Fisher exact test

## Discussion

CSE is an uncommon iatrogenic disease caused by endometrium implantation in the incision during cesarean delivery. In the present study, we investigate 198 cases of CSE over a period of 13 years, providing detailed information that helps us to better understand the clinical characteristics of this rare condition.

Several theories about the pathogenesis of AWE have been proposed, such as the implantation theory, the coelomic metaplasia theory, and the lymphatic or hematogenic dissemination theory [8, 19]. As the most common type of AWE, CSE is best explained by the iatrogenic direct implantation theory. During cesarean delivery, endometrial tissue is seeded into the wound. With an appropriate supply of nutrients and hormonal stimuli, these endometrial cells survive and proliferate, finally leading to CSE. In the present study, most of the endometriomas were located in a corner of the incision scar: 83.0% in Pfannenstiel incision scars and 84.2% in vertical midline incision scars. In another large retrospective study, conducted by Yan Ding et al., similar results were obtained [20]. In their study, 77.1% of the endometriomas were located in the corners of the scars. This is probably because endometrial cells are less easily removed from the corners of the incisions during CS. Thus, our data also support the iatrogenic cell implantation theory. However, the implantation theory alone cannot completely explain the pathogenesis of CSE, given the low incidence of CSE. Residual endometrial cell contamination of the wound during CS occurs often and sometimes is inevitable, but CSE is rare. Hereditary predisposition may confer susceptibility to the development of CSE [21].

CS is one of the most common surgical procedures performed on women worldwide. Pfannenstiel incision and vertical midline incision are the two most frequently used abdominal skin incisions. The vertical midline incision has the advantages of speed of abdominal entry and less bleeding, but has a higher risk of incisional hernia and results in a less cosmetically pleasing scar. Conversely, the Pfannenstiel incision has a lower risk of incisional hernia and results in a better cosmetic appeal. However, the Pfannenstiel incision usually involves more dissections, and the blood loss following dissection may be greater [22].

CSE is a complication of cesarean surgery. Unfortunately, the relationship between the CS incision type and the pathogenesis of CSE is still unknown. The Pfannenstiel incision is the most commonly reported type for the occurrence of CSE; however, because of the disease rarity and the need for the pathological confirmation of the diagnosis, it is difficult to estimate the population-wide incidence of CSE for different incision types [19, 20]. Demiral et al. speculated that Pfannenstiel incisions confer a higher risk of CSE than do midline incisions, but without sufficient evidence [23]. In this study, the latency period of CSE was 31.6 ± 23.9 months, which was comparable with that reported in other studies [19, 24]. However, when comparing the latency period of CSE in patients with Pfannenstiel incisions to those with vertical midline incisions, we observed a significantly shorter latency in Pfannenstiel incisions (24.0 vs 33.0 months, *P* = 0.006) (Table 3). In other words, CSE in patients with Pfannenstiel incisions occurred earlier than in patients with vertical incisions. This indicates that, compared to the vertical incision, the Pfannenstiel incision might be more favorable to the implantation and proliferation of the residual endometrial cells. We suggest two possible causes for the favorable role of the Pfannenstiel incision. First, the Pfannenstiel incision involves wider dissection planes and more gaps, rendering tissue irrigation difficult and inducing much more endometrial cell contamination [22]. The second cause is a larger nutrient supply. Due to the longitudinal pattern of the abdominal vessels and the large dissection, more capillaries are cut off during a Pfannenstiel incision than in a vertical incision, causing more blood loss. Endometrial cells require an adequate blood supply to survive in their ectopic sites, and angiogenesis plays an important role in the pathogenesis of endometriosis [25]. Therefore, more blood loss in the Pfannenstiel incision would provide a relatively rich nutritional environment for the implantation and growth of residual endometrial cells, favoring the occurrence of CSE. Consistent with this explanation, all 11 patients in this study who had multiple endometriomas had Pfannenstiel incisions. These research findings demonstrate that the Pfannenstiel incision probably carries a higher risk of CSE than the vertical midline incision. Another interesting result from this study is that deeper endometrioma locations are correlated with longer latency periods. This is probably due to the fact that the deeper endometriomas could not be easily noticed.

Although CSE is a rare event, it manifests as a painful subcutaneous mass and usually bothers the patient for several years. Additionally, CSE can undergo malignant change, which is rapidly fatal and has a survival rate of only 57% [14]. Hence, it is necessary to take precautions to prevent or reduce the occurrence of CSE. On the basis of the implantation theory, we propose a variety of measures: careful flushing and irrigating before closure; using separate needles for uterine and abdominal closure; and not using a sponge to clean the endometrial cavity following complete delivery. Extending the breastfeeding period to delay menstruation has also been proposed for preventing CSE, but without scientific corroboration [21]. In our study, 83.3% (174/209) of the scar endometriomas were located in corner sites of the wound. Therefore, the abdominal wound should be cleaned thoroughly with saline solution before closure, especially the corner sites. Additionally, endometriomas were more common in superficial parts of the abdominal wall, i.e., 12/209 (5.7%) were present in the adipose layer and 135/209 (64.6%) between the adipose layer and the fascia layer, accounting for 70.3% of the total endometriomas. Therefore, careful flushing and irrigation of the adipose layer and fascia layer during closure is critical.

All of the patients in our study underwent surgical excision for the treatment of CSE. Generally, surgical treatment offers the best chance for both making a definitive diagnosis and treating CSE. Medical therapy has a low success rate is associated with adverse effects.

As a retrospective study, some limitations in this study could not be avoided. For example, the data about the CS procedures lacked details such as the layers of closure, type of suture materials, and operation duration. These factors might also affect the occurrence of CSE. To address these questions, further studies will be required in the future.

## Conclusions

Concerning the rising CS rate, CSE may occur more frequently than generally assumed. Early diagnosis, treatment, and prevention of CSE are worthy of our attention. In our large, retrospective study, we systematically reviewed the clinical features of CSE and we provide the first evidence that the Pfannenstiel incision carries a higher risk of CSE than the vertical midline incision. The findings in this study will help us to better understand CSE and devise precautionary measures to reduce the occurrence of the disease.

## Abbreviations

AWE: Abdominal wall endometriosis
CS: Cesarean section
CSE: Cesarean scar endometriosis
